# Research on Quality Evaluation of Product Interactive Aging Design Based on Kano Model

**DOI:** 10.1155/2022/3869087

**Published:** 2022-01-31

**Authors:** Yin Liu, Weiwei Wang

**Affiliations:** ^1^Xi'an Eurasia University, EAAD School of Art and Design, Xi'an, Shaanxi 710000, China; ^2^Shaanxi University of Science & Technology, College of Art and Design, Xi'an, Shaanxi 710000, China

## Abstract

At present, China's population aging presents the characteristics of large base, fast aging process, and old age, but the research and development of product aging design in China is relatively late, which brings additional pressure to the daily life of the elderly. In such an environment, higher requirements are put forward for product aging design and service provision. Only products or services that timely meet the personalized and diversified needs of different customers can attract customers and improve customer satisfaction. How to design aging products with satisfactory appearance and humanized function is the focus of current research. Based on the product quality division of Kano model, this paper summarizes the domestic mainstream products and their functions. The designed questionnaire data are summarized; reliability and validity analysis, as well as descriptive statistical analysis, is performed on this basis for the constructed product service quality evaluation system. The service quality indicators are divided into three categories according to the Kano model: overall one-dimensional quality, charm quality, and necessary quality.

## 1. Introduction

With the refinement of management and the deepening of customer-centered management concept into enterprise management, the quality management of products and services has become a hot management issue [1]. Quality management methods and systems emerge one after another, and they have played an important role in different fields and contents. At the same time, with the deepening trend of aging in China, the number of elderly groups is increasing [1]. The educational needs of the elderly are also increasing. The popularity of smart phones and mobile Internet is affecting the daily life of the elderly. This paper collects and analyzes the relevant literature on product interactive aging design at home and abroad and finds that the current product interactive aging design in China is poor, and the product delivery design experience for the elderly needs to be improved [2]. Quality evaluation is a core part of quality management. Different quality management methods are permeated with corresponding quality evaluation methods. How to select scientific means to evaluate the service quality of enterprises in order to improve customer satisfaction and loyalty is a practical problem faced by enterprises [3]. At present, Kano model still has many limitations in practical application, and its quality evaluation method needs to be further supplemented and modified [4]. The Kano model is applied to the research of products for the elderly, mining the characteristics of products used by the elderly, and the corresponding design strategies are summarized, which is of great significance to guiding the aging design of products.

Today's environment faced by enterprises is not what it used to be. With the further interruption of monopoly industries, the market presents a strong competitive situation, which has played a significant role in promoting economic development, but at the same time, it poses severe challenges to competitive enterprises [5]. Population aging refers to the rising trend of the proportion of the elderly population in the total population under specific time and space conditions. With the increase of the number of the elderly in China, the educational needs of the elderly are becoming increasingly prominent [6]. In this environment, the interactive aging design of products is challenged. At present, many research contents focus on putting forward suggestions from the aspects of product interaction design and visual design, but there is lack of research on users' overall perception of product attributes. The overall perception research includes which attributes of the product are more important to users and which characteristics can better affect use satisfaction [7]. The introduction of Kano model into the design of aging interaction boundary of products can study the impact of different functions on user satisfaction and help designers reasonably allocate design resources. The service product itself has the characteristics of intangibility, which is more complex than the evaluation of physical products, which increases the risk in the process of consumer purchase [8]. Therefore, how to select scientific means to evaluate the service quality of enterprises in order to improve customer satisfaction and loyalty is a practical problem faced by enterprises. This paper constructs a service quality evaluation model of product interactive aging design, in order to help enterprises improve service quality and product interactive aging design.

The key to accelerating economic development is quality. There will be no real and tangible benefits unless quality is improved. Appropriate quality evaluation methods are essential for improving product quality and increasing enterprise international competitiveness [9]. To construct the nonlinear relationship between consumer satisfaction and product performance, a two-dimensional model can be used: on the one hand, it is the subjective feeling of consumers; on the other hand, it is the objective state of products. The Kano model converts specific attributes of products or services into customers' income, which is then divided into quality factors in order to analyze consumers' consumption intention and attitude [10]. As a result, the Kano model is widely used in quality control, new product development, and other areas. The rise in the number of elderly groups has heightened interest in their spiritual lives. Despite the fact that there are a lot of elderly products on the market, there are not many products specifically designed for them [11]. The current products are geared more toward teenagers, college students, and workers on the job. This paper examines the physiological and psychological characteristics, as well as the motivation and challenges of using a smartphone app by the elderly, and summarizes the pertinent information. This paper constructs a product interactive aging design quality evaluation model based on Kano model and uses Kano questionnaire to collect users' attitudes toward functions, so as to divide the functional attributes of products and rank the importance of each functional attribute. Combined with Kano model research results and interactive evaluation dimensions, the corresponding design strategies are obtained. The aging principle is verified by usability test and satisfaction evaluation, and the product interaction aging practice based on Kano model is completed.

## 2. Related Work

According to literature [12], quality means those characteristics in a product or service that can meet customer needs and make customers satisfied; it also means that a product or service is free of defects. According to literature [13], quality factors require not only a large category of judgment, but also a quantity that can effectively measure the category of quality factors in the Kano model's limitations. Only with this amount of data can a horizontal and comprehensive comparison of different products (or the same product) be made. Literature [14] proposed a Kano model-based system for evaluating personal online banking service quality and divided the service quality of personal online banking in China. According to the article, banks must improve the reliability and security of personal online banking; for one-dimensional quality, banks must improve the ease and speed of their operations and establish good communication with customers; and for charm quality, banks must improve innovation and market segmentation to improve service efficiency. According to the literature [15], customer satisfaction and perceived service quality have different relationships with expectations. A higher level of satisfaction does not imply a higher level of perceived quality. Expectations are divided into three categories: core attribute expectations, important attribute expectations, and other attribute expectations. Customers' perceived quality evaluation can be improved by meeting consumers' expectations of important attributes, and consumer satisfaction can be improved by meeting consumers' expectations of additional attributes. Customers' perceptions of service quality are multidimensional, according to literature [16], and service quality can be measured from five perspectives: reliability, responsiveness, assurance, empathy, and tangibility. Consumers' expected quality is determined by their personal needs and previous experience. A combination of four types of gaps can be used to express the gap between the quality of service and the quality expected by customers. Literature [17] believes that in the Kano model, a lot of information can be obtained by using sample data, which also plays an important supporting role for the dynamic changes of quality factors. Literature [18] proposed an I-Kano for customer satisfaction improvement decision-making, proposed Kano index and priority index, in order to use quantitative analysis methods, in the framework of the Kano model, combined with customer satisfaction, product, or service. The quality factors are judged and ranked by importance. Literature [19] applies the Kano model to engineering mechanics to determine the impact of various service qualities in the real estate industry on customer purchasing decisions. Literature [20] believes that perceived service quality includes two aspects: technical quality and functional quality. Literature [21] improved the calculation method of the Kano model and proposed a calculation method of the importance of the fuzzy Kano model. On the one hand, the fuzzy Kano questionnaire is used, and on the other hand, the demand importance adjustment function is introduced to calculate the importance of different quality attributes. This method is applied to the demand analysis of a company's combine harvester. Literature [22] proposed that customer expectation means the level expectation that customers can achieve for products or services, which is an exogenous variable. Perceived value is the perceived actual quality level, which is divided into two observation variables: the price evaluation of relative quality and the quality evaluation of relative price. Literature [23] believes that the more sufficient the service quality is, the higher it is than consumers' expectations, the more satisfied consumers will be. Literature [24] proposed an analytical Kano model on the basis of analytical customer relationship management. The model realizes the objective classification of quality factors by constructing Kano satisfaction index and importance index. They integrate consumers' important perception of each product or attribute in the questionnaire design, and finally obtain the priority to improve the service quality attribute. Literature [25] holds that when incentive factors are available, they will increase people's job satisfaction, but when they are lacking, they will not lead to people's dissatisfaction. When health care factors are available, they will eliminate people's dissatisfaction, but will not increase people's satisfaction, while when they are lacking, they will cause people's dissatisfaction. Literature [26] uses the methods of fuzzy clustering and fuzzy transformation semantics to judge the quality attributes based on Kano model and puts forward a way to obtain product personalized requirements. In this paper, Kano model is used to explore the interactive aging design of products. And it explores the rules of consumers' judgment on the quality characteristics of products or services, find out the key quality factors such as charm quality, one-dimensional quality that affect consumers' satisfaction or attract consumers' attention, and explore the correlation degree between consumers' different personality characteristics and their judgment on quality factors, so as to provide suggestions for interactive aging design of products.

## 3. Methodology

### 3.1. The Design of Product Interaction Adaptive Aging Based on Kano Model

With the development of information age, China has entered a sub-deep aging society. In order to make the decline of old people's function and follow-up study of individual ability adapt to the rapid change of product interaction information, we should reconstruct the prototype of old people's interaction, optimize the interaction mechanism of old people's products, and build the design method system of product interaction suitable for aging [27]. Therefore, the interaction of products is more suitable for the physiological and psychological characteristics of the elderly group, the interaction of the elderly users is more accurate, natural, smooth, and relaxed, and the elderly products are safer, more comfortable, healthier, and more efficient.

Aging design is a design based on the physiological and psychological needs of the elderly and their experience, taking into account the security needs and belonging needs, and at the same time trying to realize the spiritual needs and self-realization needs of the elderly to improve their quality of life [28]. The research of product interaction aging design for aged care service with Chinese characteristics supports the scientific and reasonable generation of product aging interaction design system for social aged care service from the logical level. From the technical level, the interactive design of aging products for social pension services can be quickly and effectively transformed into an applied method system.

**Figure 1:**
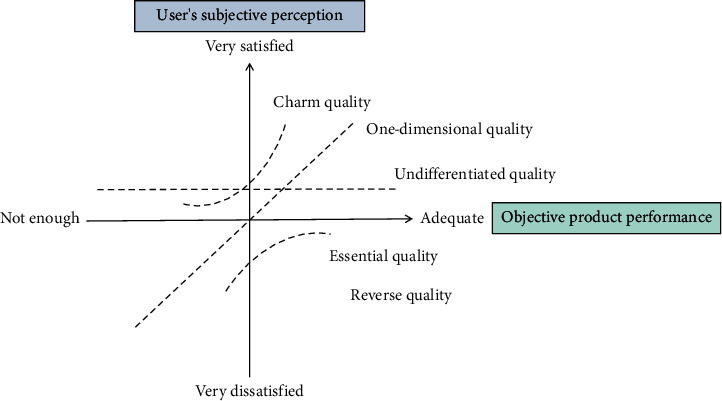
Kano model.

Product demand reflects the general requirements of users for enterprises and products and is the starting point of the whole life cycle of products. The process of product innovation and development largely depends on the understanding and analysis of product demand. Different personality consumers have different judgment results on quality factors [29]. Consumers with different personality types have different consumption preferences and habits, which makes it difficult to get satisfaction. According to the different influence modes of product attributes on consumer satisfaction, Kano model mainly classifies them into three different factors. They are charm factor, expectation factor, and necessary factor. Kano model divides users' demands into three categories: basic demands, normative demands, and interest demands. These three kinds of demands reflect the different levels of demands of users for products, and the demands will change with the changes of market and time. Interest will degenerate into normative demand, and normative demand will degenerate into basic demand. Aging products are designed and developed according to the needs of the elderly, so the products should reflect the needs of the elderly. The design elements of aging products include functional elements, aesthetic elements, safety elements, and ease of use elements.  Figure 1 shows Kano model.

With the improvement of people's cultural level and living standard, the product system that carries the entertainment and life of the elderly has been popularized to some extent, which has also laid a certain foundation for aging products. Although the types of aged products on the market at present are very single, lacking in innovation and serious homogenization in design, they cannot bring rich product experience to the aged consumers. However, from the physiological characteristics, life and behavior of the elderly, the elderly products can be roughly divided into three categories:

1. *Life Support Products*. Life support products are a common demand of the elderly. These products are designed by designers according to the specific life behavior needs of the elderly who need assistance. With these products and more professional medical care products, some elderly people can take care of themselves.
2. *Fitness Products*. The improvement of the average life span of the elderly is closely related to the increase of leisure time, increasing attention to their healthy, happy, and full life. By participating in various ball games with friends, we can increase the fun of our later life, exercise our thinking system, and prevent inattention, memory decline, and slow response caused by aging and degeneration.
3. *Leisure and Entertainment Products*. At ordinary times, the elderly will take part in some intellectual development projects because proper use of the brain can help to slow down brain degeneration and aging, prevent Alzheimer's disease, and maintain the vitality of thinking. These products are all aging products with the ultimate goal of developing the intelligence of the elderly, preventing brain degeneration by using the brain properly but not excessively, enhancing the interests and hobbies of the elderly, and increasing communication activities. To a certain extent, it can satisfy the spiritual needs of the elderly who are not satisfied with the old age, pursue health, hope to get attention and attention, and have their own hobbies.

There is a linear relationship between quality factors and customer satisfaction in the traditional quality model plough. To obtain the nonlinear relationship between customer satisfaction and product performance, the Kano model breaks through the previous one-dimensional model and uses the two-dimensional model of customer subjective feelings and objective performance of products and services. The Kano model can fully analyze product customized attributes and determine whether or not each customized attribute should be implemented and in what order. We should prioritize product customization attributes that can improve consumer satisfaction to a greater extent and make an appropriate trade-off between the manufacturer's production capacity and consumer satisfaction, in order to ensure that consumer satisfaction does not decline.

To implement the Kano model, the first step is to set up a questionnaire about product customization attributes. The results of the reliability analysis are not used to reflect the correctness of the results of the questionnaire, but to evaluate the reliability and stability of the questionnaire. Assuming that the result of the questionnaire is the algebraic sum of *K* items under investigation: *X*=*Y*_1_+*Y*_2_+⋯+*Y*_*K*_, then the Cronbach's *a*-reliability coefficient is defined as follows:

(1) $$\begin{array}{c} \alpha =\frac{K}{K-1}\left(1-\frac{\sum_{i=1}^{K}\sigma_{Y_{i}}^{2}}{\sigma_{Y}^{2}}\right). \end{array}$$

Among them, *σ*_*Y*_^2^ is the variance of the total score of the observed questionnaire test, and *σ*_*Y*_*i*__^2^ is the variance of all respondents' scores of the currently observed items.

Let domain *X*={*x*_1_, *x*_2_,…, *x*_*k*_}, that is, the collection of all functional requirements. Fuzzy set *A*={Br, Pr, Er}, *A* ∈ *F*(*X*).

There are *n* evaluation members, who are asked to independently give the estimated value *m*_*i*_(*i*=1,2,…, *n*) of *μ*_*A*_(*x*_0_) and calculate its average $\bar{m}$ and deviation *d*:

(2) $$\begin{array}{c} \bar{m}=\frac{1}{n}\sum_{i=1}^{n}m_{i},\\ d=\frac{1}{n}\sum_{i=1}^{n}\left|m_{i}-\bar{m}\right|. \end{array}$$

For a given error *ε* > 0, *μ*_*A*_(*x*_0_) can be used as the approximate value of $\bar{m}$ if the deviation satisfies *d*≤*ε*. If *d* >*ε*, repeat the above steps until satisfactory accuracy requirements are met.

When the evaluation experts estimate each membership degree, it is better to give the estimated value of *x*_1_, *x*_2_,…, *x*_*k*_ at a time than to estimate *a*_*i*_(*i*=1,2,…, *k*) individually, because the overall estimation is carried out in comparison, which makes the decision easy to make and relatively reasonable.

### 3.2. Quality Evaluation Research

Intelligentization has become the Internet age's label, and the design of aging products is following suit, with many aging products already possessing some intelligence. As the elderly's functions deteriorate, they require more outside assistance. Intelligent aging products have a certain “wisdom” that can not only reduce the elderly's physical consumption, but also minimize the difficulty of use, and realize the operation and use of the products through systematic operation. Assume the elderly group is the research object, dynamically collect their living habits and behavior patterns, and build an elderly group behavior model. Data understanding, association reasoning, clustering analysis, and other algorithms are used to extract and deconstruct the typical behavior model of the elderly group, collect physiological signal information, and mine interactive information based on objects. The output interaction information is functionally reconstructed from the perspective of interaction mechanism, and the architecture forms are cognition, understanding, decision-making, implementation, and feedback, in order to construct the interaction prototype for observation and understanding, exploration and iterative design, and communication and evaluation, according to the interaction demand.

**Figure 2:**
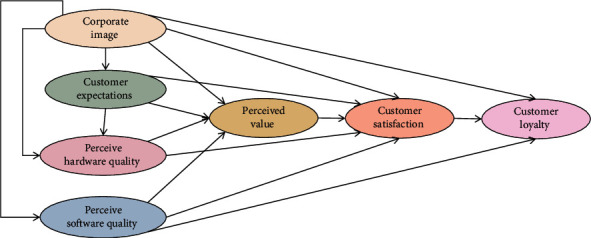
Customer satisfaction index structure model.

It is necessary for manufacturers to fully understand consumers' preferences and their information feedback on product satisfaction so as to formulate appropriate product optimization strategies and continuously improve product quality. The personality characteristics of consumers can be divided into three categories: introverted rational type, extroverted obedient type, and extroverted independent type. Among them, introverted rational consumers do not like to have too much interaction with others during the shopping process. Consumers with extroverted personality will show higher compliance with others. From the consumer's point of view, the quality of products is reflected in the degree of satisfaction of consumers.  Figure 2 shows the structural model of customer satisfaction index.

Because consumers' own demand preferences are ambiguous and their cognitive process of demand preference is uncertain, it is often difficult for nonprofessional consumers to directly judge the quality of a product. However, from the standpoint of the product, the reasonable division of product attributes allows for the visualization of consumer demand, because consumer satisfaction with products can be measured by whether, how, and how these attributes are realized, and understanding the mapping relationship between consumer demand and product attributes is critical to the demand visualization process.

The attribution degree of quality elements is actually the problem of calculating the maximum leading degree of components in the distribution vector of quality elements. Therefore, the distribution vector is directly regarded as a set of numbers to calculate the maximum lead. The calculation method of the attribute degree of quality elements is as follows:

(3) $$\begin{array}{c} \eta \langle T\rangle =\eta \langle T\left(\text{QE},t\right)\rangle ={\left(\eta^{\prime }-\psi +1\right)}^{\arctan\left(N-1+\tan\;1\right)}. \end{array}$$

Among them, the determination of the value of *ψ* can use the half-number principle and can also be determined by means or other methods. The external advantage can be characterized by the discrete coefficient of a set of values other than the maximum value. The dispersion coefficient is a measure of the uniformity of the distribution between a group of numbers, which has the same points as the external advantages discussed in this article.

If we define a product attribute set *F*={*f*_1_, *f*_2_,…, *f*_*i*_,…, *f*_*q*_} that includes *q* product attributes, and a surveyed person set *E*={*e*_1_, *e*_2_,…, *e*_*j*_,…, *e*_*p*_} that includes *p* respondents, then, for each attribute, there are

(4) $$\begin{array}{c} e_{j}⟶i=\left(a_{j⟶i}^{+},a_{j⟶i}^{-}\right)\;i=1,2,\ldots ,q;\;j=1,2,\ldots ,p,\\ a_{j⟶i}^{+},a_{j⟶i}^{-}\in \left\{{\;}^{\prime \prime }{\text{Like}}^{\prime \prime },{\;}^{\prime \prime }\text{Must}-{\text{be}}^{\prime \prime },{\;}^{\prime \prime }{\text{Neutral}}^{\prime \prime },{\;}^{\prime \prime }{\text{Live with}}^{\prime \prime },{\;}^{\prime \prime }{\text{Dislike}}^{\prime \prime }\right\}. \end{array}$$

Among them, *a*_*j*⟶*i*_^+^ and *a*_*j*⟶*i*_^−^ are the answers to the positive and negative attribute questions, respectively.

Behavior mode is the foundation of product interaction mode, and product interaction mode is the result of behavior mode, which is interactive and interrelated. Different behavior mode information feedback determines different interaction modes, and different behavior mode information feedback determines different interaction modes. People-oriented user needs are prioritized in interactive design efforts to create and build meaningful relationships between people, products, and services. Interactive aging product design can improve elderly consumers' experiences with aging products, help them accept new things, and thus improve their quality of life. This paper investigates the relationship between elderly behavior patterns and product interaction based on a prototype of elderly interaction. The premise is that there is a mapping relationship between the physiological information of the elderly and product interactions, including the mapping content and structure. Its core is the old age behavior pattern's action mechanism on product interaction; its essence is the old age behavior pattern's support for product interaction and the reverse verification of old age behavior pattern by product interaction.

Define the transformation rule *T* and the frequency index *C*_*i*_(*k*):

(5) $$\begin{array}{c} \text{count}\left(T\left(e_{j⟶i}\right)\right)\equiv 1,\\ C_{i}\left(k\right)=\overset{p}{\underset{j=1,T\left(e_{j⟶i}\right)=k}{\sum }}\text{count}\left(T\left(e_{j⟶i}\right)\right),\\ K\left(f_{i}\right)\in \left\{k^{\prime }|C_{i}\left(k^{\prime }\right)=\underset{\forall k}{\max}C\left(k\right)\right\},\\ T\left(e_{j⟶i}\right),k,k^{\prime }\in \left\{A,O,M,I,R,Q\right\}. \end{array}$$

Among them, *K*(*f*_*i*_) is the Kano classification result of product attribute *f*_*i*_. That is to say, by summing up the regional statistical percentages of each factor of a certain product attribute, the factor corresponding to the maximum value is the factor that the attribute is divided into.

The technology and environmental conditions provided by the mobile Internet make it possible to collect real-time and comprehensive data for the elderly. By using cloud processing and other means, it can not only reduce the functional burden of products, but also provide digital information services based on similar people. In the future, a big data information platform will be established for the elderly, which will provide conditions and entrances for the intervention of various comprehensive services. At the same time, the interactive experience based on communication will be realized through the construction of home or community networks, which will also provide the possibility for the optimization and upgrading of system services.

## 4. Results Analysis and Discussion

Kano model is based on customers' perceived quality. Kano model keeps approaching customers' expectations by developing charm quality characteristics, and its purpose is to maximize customers' potential needs. Kano model can be used to accurately find out the root causes of service quality problems, which is helpful for enterprises to prescribe the right medicine and formulate correct development strategies. Existing demand importance evaluation methods have obvious flaws when it comes to dealing with the importance issue. Because a large number of complicated and trivial needs of users are asked, it is easy to bore users and lead to incomplete and inaccurate inquiry results when using the user inquiry survey method. Although using the repetition frequency of users' requirements can avoid the drawbacks of the previous method, judging the importance solely on the basis of the repetition frequency of users' requirements is unreliable. Interest demand is a very rare occurrence in the Kano model's user demand acquisition process, but it is extremely important product demand information.

**Figure 3:**
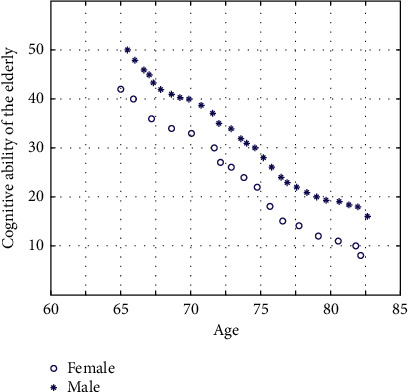
Trend of cognitive ability of the elderly with age.

In the daily life of the elderly, cognitive ability is an important ability for the elderly to receive information about external things. The decline of cognitive ability of the elderly not only brings inconvenience to their own lives, but also troubles the lives of other family members. At the same time, the physiological function of the elderly is degraded due to the increase of age, as shown in Figure 3, it is physically and psychologically difficult to keep up with the fast-paced modern life.

The data of Kano questionnaire on product service quality are preliminarily summarized, and then the related quality characteristics are classified by Kano attributes. The priority order of attributes at the same level is determined by calculating the better-worse index. The value of better is usually positive, which means that if a certain attribute is provided, the user satisfaction will be improved. The larger the positive value, the stronger the effect of improving the user satisfaction, and the faster the increase. The value of worse is usually negative, which means that if a certain functional attribute is not provided, the user's satisfaction will decrease. The more negative the value is, the stronger the effect of decreasing the satisfaction will be and the faster it will decrease.

**Figure 4:**
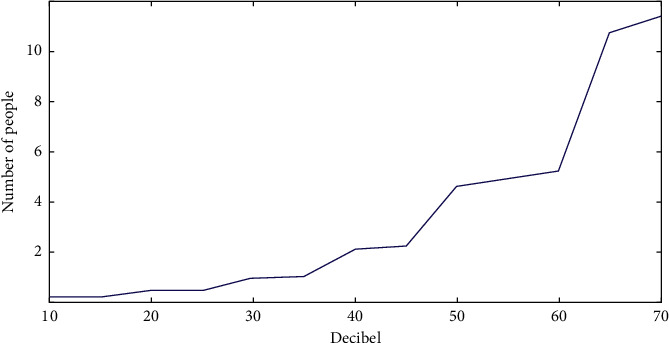
Minimum sound threshold statistical result.

In this paper, the lowest threshold of hearing perception of elderly users is studied experimentally. The test mainly focuses on the sound threshold test of elderly users, and the sensitivity of elderly users to sound is found. When the elderly users are using the product, the sound prompt with appropriate volume can attract the attention of the elderly users so that the elderly users can grasp the state of the product. As shown in Figure 4, the sound threshold that the elderly users can distinguish is obtained, and the sound feelings that the elderly users hear are listed, which provides the data source for the research of product interactive hearing below.

The statistical data of sound threshold can be used to conclude that the elderly users' hearing loss is severe. The elderly users' auditory attention is hardly drawn to sound stimulation with a volume below 40 dB. The elderly users can only fully receive the sound stimulation and recognize the sound content when the volume is above 40 dB. As a result, in interactive aging product design, the sound feedback prompt of products must be above 40 dB in order for elderly users to recognize and master the product status.

On the one hand, the Kano model analysis results reflect the user's preference, which helps manufacturers gain a better understanding of their customers' individual needs and, ultimately, determine the customizable product attributes that must be entered into the aided design support system. On the other hand, it has the potential to improve the configuration process and efficiency. Consumers and manufacturers have formed a benign closed loop around their individual needs under this framework, from the perspective of consumers. Kano analysis, which improves the efficiency of product configuration in the CAD support system, fully reflects the collaborative value of consumer demand. He directly participates in the product configuration process as an individual consumer, efficiently obtains a personalized product design scheme through human-computer interaction, and fully satisfies his own preferences.

**Figure 5:**
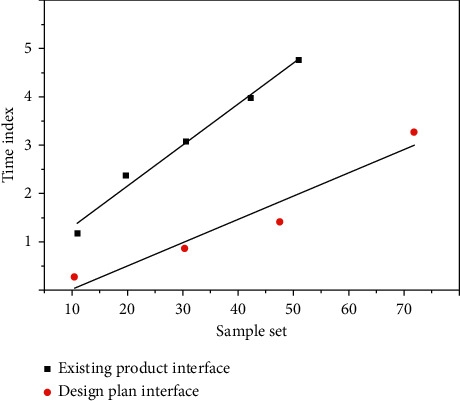
Average test time for two interfaces.

The visual effect and font size of the interface will affect the cognitive efficiency of the elderly users. In order to verify the feasibility of the operation interface of this scheme and whether it can meet the needs of the elderly users for easy identification and operation, the usability test of the operation interface is carried out. Using the software to make dynamic effects, key operation, feedback prompts, and so on, we simulate the operation mode of the old users' real use of the rice cooker and select an existing operation interface of the rice cooker in the market for comparative test. The experimental results are shown in Figure 5.

It can be seen from Figure 5 that the average time for the elderly users to operate the designed interface is less than that of the existing interface. The design scheme is obviously superior to the existing product scheme, benefiting from concise interface interaction, good human-machine dimension of parts, and appropriate feedback information. This also verifies the effectiveness of the aging design principle. The test of this design scheme relies on software to simulate the actual operation effect, and the final test result is convincing to a certain extent. The actual product interaction effect also needs real products as the evaluation object.

**Figure 6:**
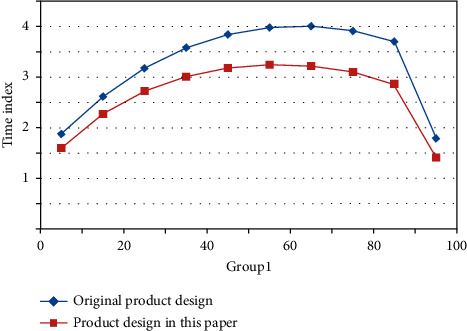
Time for single operation of the first group of users.

**Figure 7:**
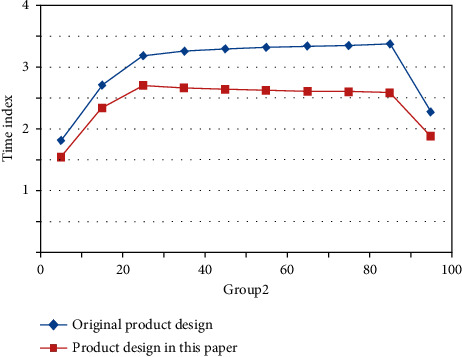
The second group of users' single operation time.

By refining the common factors that can represent the overall structure of the questionnaire, the cumulative explanatory quantity in the analysis results can explain to what extent the test questionnaire can reflect the true characteristics of the measured object; that is, it can test the structural validity of the questionnaire. Based on the two tests before and after the innovation activity, the transition matrix is constructed by analyzing the changes of a certain quality element among five categories of quality elements. Assuming that the innovation effect is stable, the stable state of Markov chain can indicate the degree of innovation. Figures 6 and 7 reflect the time taken by users to evaluate individual product design schemes in each generation population under the two methods.

According to the comparison results, the method proposed in this paper effectively reduces the user's operation time, and both the average time for evaluating each generation population and the total time for completing a product customization are less than the time required by traditional methods. Therefore, the method proposed in this paper can fully alleviate user fatigue in the process of product customization.

Although the importance analysis method for structural requirements is the same as for functional requirements, the distribution of weight values differs. Because of their complaints and dissatisfaction with the original product structure and behavior, users, for example, pay more attention to basic needs. If these factors are not adequately considered in the design of new products, user dissatisfaction will skyrocket, and the image of new products will suffer. As a result, businesses should adjust the criterion layer's weight set in accordance with their own product development strategies. Before the experiment, the data were set and recorded, and the behaviors and actions of the subjects using the products were coded to ensure the reliability and validity of the final data. The classification of behaviors should be complete and nonoverlapping, and behaviors should be clearly defined so that observation is not made more difficult or inconsistent. The behaviors in this experiment are grouped based on this requirement.

**Figure 8:**
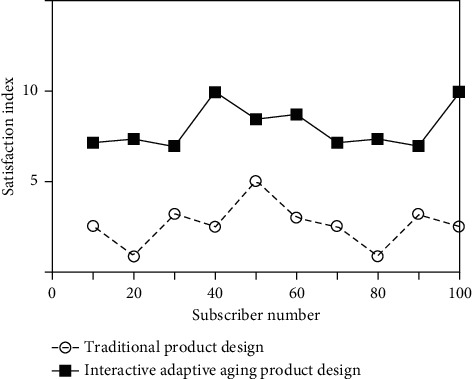
Comparison of product design satisfaction evaluation.

After the product design scheme is completed, in order to verify whether the design scheme can be accepted by the elderly users, the elderly users need to evaluate the product and obtain their satisfaction with the design scheme. This evaluation is mainly conducted from three aspects: product appearance, interface effect, and operation mode. Make Likert scale from the aspects of product appearance, usability, identifiability, interface layout, and operation mode.  Figure 8 is a comparison of satisfaction evaluation between traditional product design and interactive aging product design for elderly users.

It can be seen from Figure 8 that typical elderly users are satisfied with the design scheme of this product. The average satisfaction of product appearance, usability, identifiability, interface layout, and operation mode is generally high. The scientific design of this paper is verified.

The characteristics of quality elements are examined in depth in this chapter. In terms of time characteristics, charm, expected quality, and basic quality sink, from the perspective of enterprises, attractive quality is a pursuit goal, in which enterprises hope that the quality and physical characteristics of their products can become attractive quality to customers. The influencing factors of service quality are studied from the perspective of consumers' types, in combination with the characteristics of the elderly population and consumers' personality in the questionnaire survey results. To investigate the possible rules between different personality characteristics and Kano model quality judgment, summarize the consumers' Kano quality judgment results separately.

## 5. Conclusions

Fundamentally improving the interactive performance of the aged products, making the interaction between the aged users and the products more accurate and relaxed, and making the aged products safer, more comfortable, and more efficient, as well as improving the inclusiveness and interactivity of the hardware facilities for the aged groups under the aged care model with Chinese characteristics, and ensuring the level and quality of age care are of strategic and practical importance. The goal of designing products for the elderly is to provide a positive product experience based on barrier-free use so that product usage is more in line with the people-oriented concept and a high-quality user experience, promoting the stability and prosperity of a harmonious society. Product design, on the other hand, is an important aspect for businesses to remain evergreen. This paper develops a dynamic quality factor prediction model based on a thorough understanding of the characteristics of quality factors. It also introduces a brand-new method for assessing the impact of quality innovation activities. This paper proposes to evaluate the effect of interactive aging design of products using relevant Kano model theories from the perspective of the elderly. This method of assessment is more scientific and objective. At the same time, the evaluation model presented in this paper is straightforward and simple to implement.

## Data Availability

The data used to support the findings of this study are included within the article.
